# DNA methylation changes in ovarian cancer are cumulative with disease progression and identify tumor stage

**DOI:** 10.1186/1755-8794-1-47

**Published:** 2008-09-30

**Authors:** George S Watts, Bernard W Futscher, Nicholas Holtan, Koen DeGeest, Frederick E Domann, Stephen L Rose

**Affiliations:** 1 Department of Medical Pharmacology, College of Medicine, University of Arizona, Tucson AZ 85724 USA; 2 Department of Pharmacology and Toxicology, College of Pharmacy, University of Arizona, Tucson AZ 85724 USA; 3 Arizona Cancer Center, University of Arizona, Tucson AZ 85724 USA; 4 Department of Obstetrics and Gynecology, Free Radical & Radiation Biology Program, University of Iowa, Iowa City IA 52242 USA; 5 Department of Radiation Oncology, Free Radical & Radiation Biology Program, University of Iowa, Iowa City IA 52242 USA; 6 Department of Obstetrics and Gynecology, University of Wisconsin, Madison WI, 53792, USA

## Abstract

**Background:**

Hypermethylation of promoter CpG islands with associated loss of gene expression, and hypomethylation of CpG-rich repetitive elements that may destabilize the genome are common events in most, if not all, epithelial cancers.

**Methods:**

The methylation of 6,502 CpG-rich sequences spanning the genome was analyzed in 137 ovarian samples (ten normal, 23 low malignant potential, 18 stage I, 16 stage II, 54 stage III, and 16 stage IV) ranging from normal tissue through to stage IV cancer using a sequence-validated human CpG island microarray. The microarray contained 5' promoter-associated CpG islands as well as CpG-rich satellite and Alu repetitive elements.

**Results:**

Results showed a progressive de-evolution of normal CpG methylation patterns with disease progression; 659 CpG islands showed significant loss or gain of methylation. Satellite and Alu sequences were primarily associated with loss of methylation, while promoter CpG islands composed the majority of sequences with gains in methylation. Since the majority of ovarian tumors are late stage when diagnosed, we tested whether DNA methylation profiles could differentiate between normal and low malignant potential (LMP) compared to stage III ovarian samples. We developed a class predictor consisting of three CpG-rich sequences that was 100% sensitive and 89% specific when used to predict an independent set of normal and LMP samples versus stage III samples. Bisulfite sequencing confirmed the NKX-2-3 promoter CpG island was hypermethylated with disease progression. In addition, 5-aza-2'-deoxycytidine treatment of the ES2 and OVCAR ovarian cancer cell lines re-expressed NKX-2-3. Finally, we merged our CpG methylation results with previously published ovarian expression microarray data and identified correlated expression changes.

**Conclusion:**

Our results show that changes in CpG methylation are cumulative with ovarian cancer progression in a sequence-type dependent manner, and that CpG island microarrays can rapidly discover novel genes affected by CpG methylation in clinical samples of ovarian cancer.

## Background

Ovarian cancer remains the most deadly gynecologic malignancy. There were an estimated 22,430 new cases and 15,280 deaths in the United States in 2007[1]. In contrast to other gynecologic malignancies, 81% of ovarian cancers are late stage III or IV at the time of diagnosis, implying upper abdominal or distant metastases[1]. Because of late stage at diagnosis, the 5 year survival of ovarian cancer for all stages is only 45%, and the 5 year survival for patients with stage III or IV disease is only 30%[1].

Aberrant DNA methylation at CpG islands, often in close proximity to transcription start sites, is associated with the epigenetic regulation of genes through altered transcription factor binding and chromatin structure[2]. Widespread changes in CpG island methylation have also been associated with neoplastic progression [3-5]. Previous researchers have successfully used various genome-wide scanning approaches to analyze tumor cells and their epigenomic states [6-10]. Results have shown that CpG islands display tumor-specific patterns of aberrant methylation[6], while selected CpG islands have been reported to show stage-specific patterns of aberrant methylation [11-15], and others have not[16]. Two recently published studies have examined broad epigenetic changes in ovarian cancer[10,11]. Widschwendter et al. examined DNA hypomethylation at satellite sequences and found a strong association between satellite DNA hypomethylation and advanced tumor stage and grade. In addition, satellite DNA hypomethylation was found to be an independent marker of prognosis. Wei et al. used CpG-island microarrays to analyze stage III and IV ovarian tumor samples and developed a classifier that predicted six month progression free survival with 95% accuracy and specificity. Neither of these studies performed a study of CpG island methylation across all stages of ovarian cancer as reported here.

We have used a 6,560 element CpG island microarray to analyze the epigenomic profile of clinical samples of ovarian tissues ranging from benign to late stage tumors. We identified CpG methylation changes that progressed with disease severity and distinguished stage III ovarian cancer from normal or LMP ovarian tissue. We confirmed the microarray results with bisulfite sequencing, 5-aza-2'-deoxyazacytidine re-expression, and correlation with previously published expression profile data.

## Methods

### Sample Collection and Nucleic Acid Isolation

Ovarian tissue samples were obtained from The University of Iowa Gynecologic Oncology Tumor Bank. Samples were collected with informed consent in accordance with the standards of the Institutional Human Subjects Protection Review Board and were surgically staged according to FIGO staging guidelines. Tumor samples were taken from primary tumors only, with no prior exposure to chemotherapy. Normal tissue and tissue samples of low malignant potential were also collected for comparison to tumor. Tissues were snap frozen at the time of surgery in liquid nitrogen and high molecular weight tumor DNA extracted with Trizol reagent by following the manufacturer's instructions (Life Technologies, Inc., Gaithersburg, Maryland). The DOT quantitative test was used to estimate the DNA concentration[17].

### CpG island library, probe preparation, microarray production

The CpG island clones, their preparation for printing, and microarray production were performed as previously described[18].

### Target preparation

Genomic DNA was cut be MseI (New England Biolabs, Beverly, MA), and then a catch-linker was ligated to the MseI fragments. The fragments were then cut with a methylation-specific restriction enzyme, McrBc (New England Biolabs, Beverly, MA). Mock-cut reference samples were exposed to the same conditions and reagents as the digested samples; however, no GTP was added to drive the restriction digest. Twenty nanograms of the mock-digested or twenty nanograms McrBc-cut genomic DNA was then amplified by PCR using primers specific to the linkers and purified with the QIAquick PCR purification kit (Qiagen, Valencia, CA). Fluorescent Cy3 or Cy5 dye was incorporated into the PCR product using the BioPrime DNA labeling system (Invitrogen, Carlsbad, CA).

### CpG Island Microarray

Two-color fluorescence hybridizations analogous to expression microarrays were used to compare DNA digested with the methylation-specific enzyme McrBc to mock-digested reference DNA as described previously[18]. After labeling, cut and mock reactions are mixed and re-purified with the QIAquick PCR purification kit. After purification, the labeled target was lyophilized to dryness, re-suspended in 60 microliters Oligo Hyb Buffer (The Gel Company, San Francisco, CA) and denatured by boiling for ten minutes. The sample was then added to the processed array slide in a chamber of the ArrayBooster Hybridization Station (Advalytix, Concord, MA). An AdvaCard with two mixer chips (Advalytix, Concord, MA) was used to cover and seal the array during hybridization at 42°C for 4–8 hours. Following hybridization, slides were washed by placing them into 50 ml conical tubes containing 2 × SSC, 0.1% SDS for five minutes, 0.06 × SSC, 0.1% SDS for five minutes, and 0.06 × SSC for five minutes all at room temperature. Slides were dried by centrifugation at 500 × g for one minute and scanned for Cy3 and Cy5 fluorescence using an Axon GenePix 4000 (Axon Instruments, Foster City, CA).

### Data Analysis

The data from scanned microarray images were extracted using GenePix software. Median pixel intensity of each spot was used for analysis. To normalize Cy3 and Cy5 signal intensities we used the "interactive linear regression" approach with minor modifications[19]. Raw data was normalized using unmethylated mitochondrial[20] sequences dispersed across the microarrays: first, all intensity values were log transformed, than linear regression was performed using data from the mitochondrial sequences only. Residuals were calculated and outliers (those residuals where |e| > 2 × standard deviation of e) were removed and the regression function was recalculated. If the difference between the r-squared values of the new and previous regression line was less than 0.001, then no further residuals were removed. Y-Intercept values were applied as correction factors to the log transformed channel 2 values of all clones. The result is that the function of log channel 1 and log channel 2 of mitochondrial clones closely approximates y = x. Data was loaded into BRB ArrayTools v3.5.0 for normalization between arrays and analysis. Representative M versus A plots for three hybridizations are shown in Additional File 1. Samples were labeled as normal, LMP, or stage III for class prediction using the Prediction Analysis of Microarray (PAM) classification algorithm, with separate training and test sample sets as recommended by Simon et al. [21]. The PAM algorithm as implemented in BRB ArrayTools was used to create linear combinations of loci and weight them according to the stability of their methylation profile within each group of samples (normal/LMP or stage III). The PAM algorithm then attempted to identify the smallest list of loci with the highest prediction accuracy as measured by 10-fold cross-validation.

Class prediction using five other methods was performed in BRB ArrayTools using sequences univariately significant at p < 0.001. Only data from the training set was used to develop the classifier. Cross-validation was done using the leave-one-out method and repeating the entire analysis for each iteration of the cross-validation including determination of which sequences were univariately significant. The permutation p-value for the cross-validated mis-classification rate was estimated by analysis of 2000 permutations in which the sample labels were scrambled and the class prediction analysis performed. The permutated p-value is the proportion of the random permutations that gave as small a cross-validated misclassification rate as was obtained with the real class labels. Additional information on how BRB ArrayTools implements the algorithms and performs cross-validation can be found in the manual: . All other settings were default.

### NKX2-3 Bisulfite sequencing

Five micrograms of genomic DNA was modified with sodium bisulfite as previously described[22]. The NKX2-3 CpG islands were amplified from the bisulfite-modified DNA by two rounds of PCR using nested primers: Forward Primer 1: 5'-GTGGTTTTGATGATGTTATTAA-3', Reverse Primer 1: 5'-ACTCCCTTACAAATACCTAC-3', Forward Primer 2: 5'-AGTTAAAGATATTTTGAATTTGGA-3', Reverse Primer 2: 5'-CTAACAAAATCTATAAACTATTTAT-3'. Both rounds of PCR were performed under the same parameters, with 1% of the first round PCR product serving as the template in the second round of PCR. PCR amplification was performed in an MJ thermal cycler (PTC200) under the following conditions: 94 C for four min followed by five cycles of 94 C for one min, 56 C for two min, 72 C for three min, then 35 cycles of 94 C for 30 s, 56 C for two min, 72 C for 1.5 min, and a final extension of 72 C for six min.

The resulting PCR product was cloned into a TA cloning vector according to the manufacturer's instructions (pGEM-T-Easy cloning kit, Promega, Madison, WI). Forty-seven positive recombinants from each of ten samples (five normal ovaries, and 5 stage III or IV ovarian tumors) were isolated using a Qiaprep Spin Plasmid Miniprep kit (Qiagen, Valencia, CA) according to the manufacturer's instructions and sequenced on an ABI automated DNA sequencer by the Genomic Analysis, Technology, & Sequencing Core at the University of Arizona. The methylation status of individual CpG sites was determined by comparison of the sequence obtained with the known target sequence. The number of methylated CpGs in each sample was counted and averaged over each tissue class. The means for normal and tumor tissue were compared using an unpaired Welch's t-test.

### Cell culture and drug treatment

ES2 cells were maintained in McCoy's media at 37 C under 95%/5% air/CO_2 _atmosphere with 10% fetal bovine serum and 100 mg/mL penicillin and streptomycin. OvCar 3 cells were maintained in the same conditions as ES3 except the media was RPMI 1640. 5-aza-2'-deoxycytidine (Sigma-Alrich, St. Louis, MO) was added to the culture media at a concentration of 0, 2, or ten micromolar for six days with drug added every other day starting on day one. On day six cells were washed with 4 C phosphate buffered saline and collected for RNA isolation using Qiagen's RNeasy Midi kit according to manufacturer's instructions.

### ENKX2-3 Real Time RT-PCR

PCR amplification was performed using Taqman primer/probes specific for NKX2-3 obtained from Applied Biosystems (Foster City, CA); the primer probe sequences are available upon request. PCR was performed with the ABI Prism 7000 sequence detection system following Applied Biosystem's PCR Master Mix protocol. Real-time PCR was carried out in triplicate on five nanograms of cDNA using parameters recommended by Applied Biosystems. Relative expression was determined by applying the comparative C_t _method, as described previously[23].

## Results

### DNA methylation differences in benign and malignant ovarian tissue

137 samples of benign and malignant ovarian tissues (ten normal, 23 low malignant potential, 18 stage I, 16 stage II, 54 stage III, and 16 stage IV) from various stages and histopathologies were analyzed to develop a broad view of DNA methylation changes with progression of ovarian cancer (Additional File 2). The CpG island microarray contained 6,560 CpG-rich genomic clones, representing approximately 15% of the CpG islands in the genome[24]. The CpG islands spanned the genome with varying proximity to 5' promoter regions: rank 1 were within 0.5 kb of transcription start, rank 2 were within 1 kb of transcription start, rank 3 were within 2 kb of transcription start, and rank 4 were located >2 kb from transcription start. Clones containing satellite repeats or >25% Alu sequence were designated as such[18].

To examine the relationship between tumor stage and changes in DNA methylation, samples were grouped by stage, and analysis of variance (ANOVA) was performed. 2,042 CpG-rich clones with significant differences in methylation by stage at p = 0.01 (Benjamini Hochberg False Discovery Rate (FDR) adjusted) were identified, and the list reduced to 659 clones by selection for at least a 1.5-fold difference in methylation between any two stages (Additional File 3). The fold change cut-off was used in addition to statistical significance to limit the number of CpG-rich sequences to those with the largest differences.

**Figure 1:**
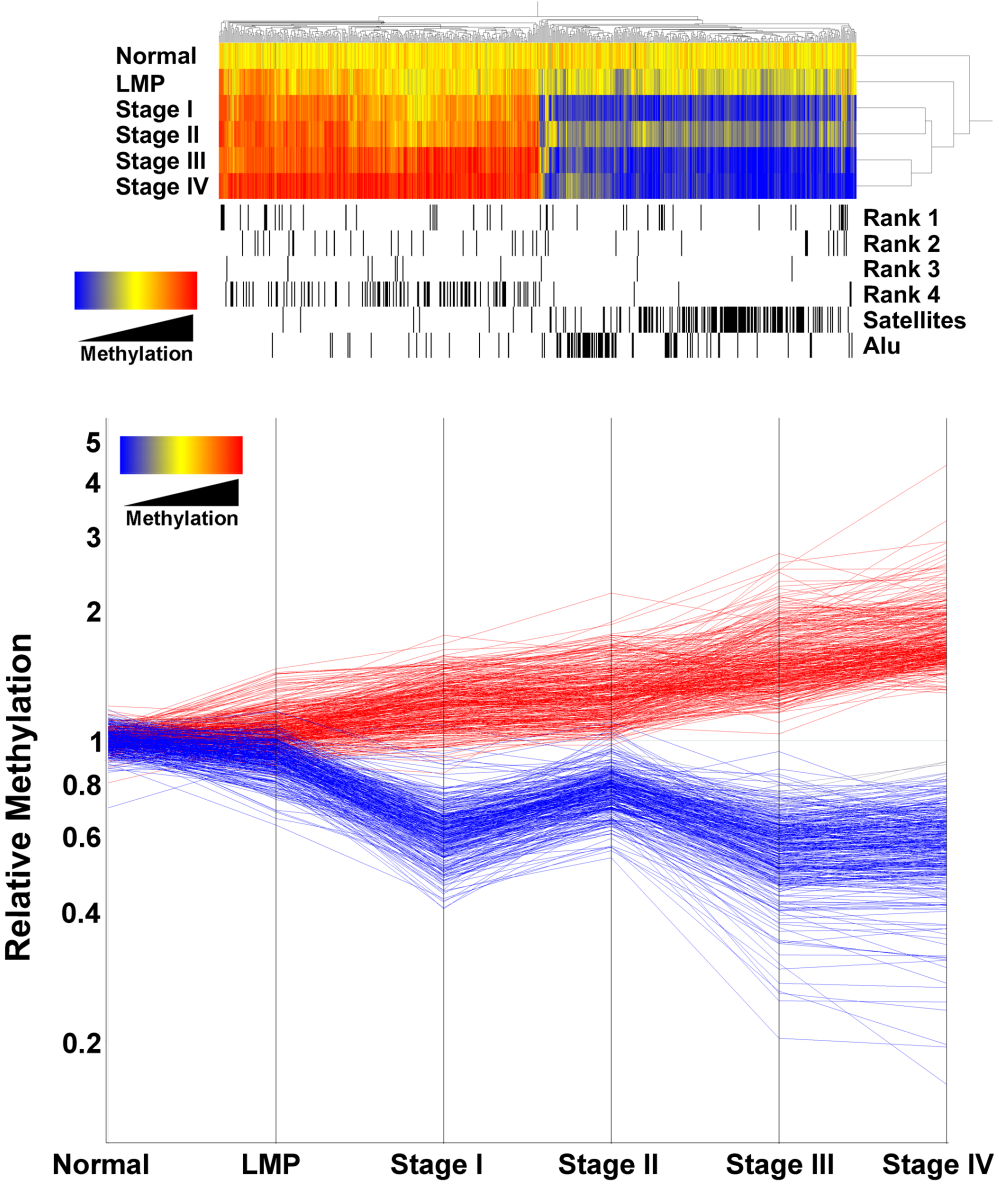
**Differences in DNA methylation patterns across the genome distinguish ovarian tissues by stage (see AdditionalFiles**2**and**3**for sample histopathology and the list of sequences used in clustering).** a) The 659 CpG-rich clones with differential methylation between any two tumor stages were used in hierarchical clustering by Pearson correlation and average linkage. Each CpG-rich clone is represented by one bar and colored by their average methylation relative to the median of the ten normal samples; hypermethylation is shown in red and hypomethylation shown in blue. Horizontally aligned black bands mark the position of specific ranks of clones: Rank 1, the clone sequence lies within 0.5 kb of the transcription start of a known gene; Rank 2, the clone lies within 1 kb of transcription start; Rank 3, the clone lies within 2 kb of transcription start; Rank 4, the clone lies >2 kb from transcription start; Satellites, probes containing satellite repeats; Alu, probes of which the clones consist of >25% Alu sequence. b) The same 659 CpG-rich clones are graphed by tumor stage. Each CpG-rich clone is represented by one line. Lines are colored by their average methylation in Stage IV; blue indicates loss of methylation, and red indicates gain of methylation, relative to the median of the ten normal samples.

The 659 CpG-rich clones were clustered along with disease stage using hierarchical clustering with Pearson correlation and distance measured by average linkage. The resulting dendrogram showed gains and losses of DNA methylation at specific CpG-rich clones were correlated with ovarian cancer progression from normal to stage IV disease (Figure 1a). 334 CpG-rich sequences gained methylation with disease progression, while 325 lost CpG methylation. The majority of CpG-rich sequences that lost methylation during cancer progression were repetitive elements (satellite and Alu sequences, Figure 1a), while those that gained methylation were primarily CpG-islands associated with gene promoters (Rank 1–4, Figure 1a). A graph of the 659 CpG-rich clones by stage showed that unlike the methylated sequences, loss of methylation was not incremental with each stage progression. While stage I tumors had lost methylation relative to normal and LMP samples, the same sequences were partially methylated in stage II tumors, and reverted back to a un-methylated state in stage III and IV (Figure 1b). Examination of the 659 sequences by histopathology and stage showed that the re-methylation of sequences in stage II tumors occurred in tumors of endometrial and serous histopathologies, but not mucinous tumors (Additional File 4). Overall, the results showed an association between disease stage and progressive hyper-methylation of promoter-associated CpG islands and hypo-methylation of repetitive sequences. The same association was seen with disease grade (Additional File 5).

### Classification of normal or LMP and stage III samples using CpG methylation data

The majority of ovarian tumors are stage III at the time of diagnosis. Based on the sample cluster results, we tested whether the cumulative CpG methylation changes could distinguish between normal or LMP ovarian tissue and stage III cancer. The normal, LMP, and stage III samples were randomly and evenly divided into two groups. The first group of 43 samples was used as a training set to develop a classifier of CpG-rich sequences to differentiate normal or LMP from stage III samples. The second group consisting of 44 samples was used to test the performance of the classifier on an independent data set. The Prediction Analysis of Microarrays (PAM) algorithm as implemented in BRB-ArrayTools was used to develop a classifier using all CpG-rich sequences on the microarray. The PAM algorithm optimizes the size of the classifier such that the fewest number of elements are included without decreasing performance.

**Figure 2:**
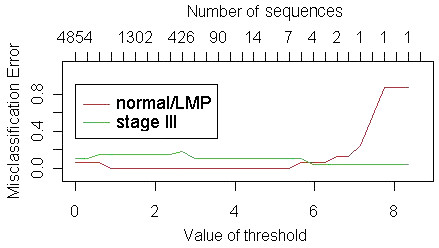
**The optimal number of sequences to predict normal or LMP ovarian tissue from stage III ovarian tumor.** Graph of misclassification rate for each class (normal/LMP tissue or stage III ovarian tumor) as a function of the threshold value in the PAM algorithm. As the threshold parameter increases, the number of sequences in the classifier decreases. The optimal point was reached at a threshold value of 6.26, or 3 sequences.

**Figure 3:**
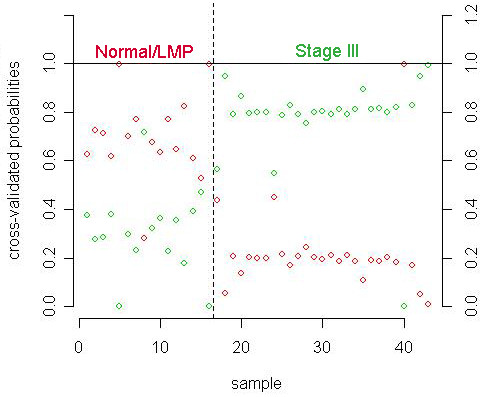
**Performance of the 3-sequence classifier during cross-validation.** The probability of each sample belonging to one class (normal or LMP tissue, red; stage III ovarian tumor, green) is shown on the y-axis for each of the 43 samples used to develop the 3-sequence classifier. One normal sample and one stage III ovarian tumor were misclassified resulting in a misclassification rate of 5%.

The PAM algorithm produced a classifier at a threshold value of 6.26 that consisted of three CpG-rich clones, all of which were hypomethylated in stage III tumors relative to normal or LMP tissue (Figure 2). The three hypomethylated sequences included two satellite sequences and one Alu repeat (clone IDs: BF.44.F11, BF.44.B7, BF.21.B1; average methylation ratios for all clones are in Additional File 6), and all three were present in the 659 sequences identified above as significant by disease stage. During cross-validation of the classifier on the training set, the three-sequence classifier had 94% sensitivity and 96% specificity. One LMP and one stage III sample were incorrectly classified (sample IDs 6 and 69, Additional File 2). Overall, the classifier misclassification rate was 5% (Figure 3). On the independent test set of samples the three-sequence classifier had a sensitivity of 87% and specificity of 100% – two normal and two LMP samples were misclassified as stage III (sample IDs 62, 64, 72, 77, Additional File 2).

A separate class prediction analysis was performed in which the training and test sets were analyzed using several class prediction methods implemented in BRB ArrayTools. The training set was used to select sequences that were differentially methylated between the classes at the p < 0.001 significance level, and resulted in a classifier with 911 sequences. The 911-sequence classifier was then tested using five class prediction methods: compound covariate predictor, diagonal linear discriminant analysis, 1-nearest neighbor, 3-nearest neighbors, and support vector machines. Cross-validation using the leave-one-out method was performed to determine the mis-classification error rate of each class prediction method. To prevent bias in estimation of the mis-classification error rate, the entire analysis was repeated during each iteration of the cross-validation, including determination of which sequences were significant on the reduced training sample. The sensitivity and specificity of the class prediction methods in cross-validation were nearly identical: 94% and 89% respectively for the compound covariate predictor, diagonal linear discriminant analysis, and 1-nearest neighbor methods; and 94% and 93% for the 3-nearest neighbors and support vector machines methods (Additional File 7). The class prediction methods were further validated by estimation of the permutation p-value of the cross-validated mis-classification rate. The entire analysis was repeated 2,000 times with scrambled sample labels to determine the proportion of the random permutations that gave as small a cross-validated misclassification rate as was obtained with the real class labels. Results showed all five class prediction methods had a permutation p-value for the cross-validated mis-classification rate of p < 0.001. Finally, strong additional support for the classifier came from the independent test set of data. When used to predict the class of the samples in the independent test set, three of the class prediction methods (1-nearest neighbor, 3-nearest neighbors, and support vector machines) were 100% correct, while the remaining two methods were 98% correct; the compound covariate predictor and diagonal linear discriminant analysis methods both mis-classified sample 73 as Stage III when in fact it was a LMP sample (Additional File 7).

Of the 911 sequences with significant (p < 0.001) methylation differences between the normal and LMP samples and the Stage III cancers, 373 had a greater than 1.5-fold change (Additional File 8). The 911 sequences used by the class prediction methods included the three sequences in the classifier developed by the PAM algorithm as well as 425 of the 659 (64%) of the sequences identified above as significant by stage with a 1.5-fold change in methylation. The large difference in the number (911) of sequences with significant differences between the classes and the three sequences in the classifier developed by the PAM algorithms reflected the fact that while a large number of sequences had altered CpG methylation between normal or LMP tissue and stage III tumor (compare normal and LMP to Stage III in Figure 1), the optimal number of sequences for good performance in class prediction was small.

### Bisulfite sequencing confirmation of CGI microarray data

**Figure 4:**
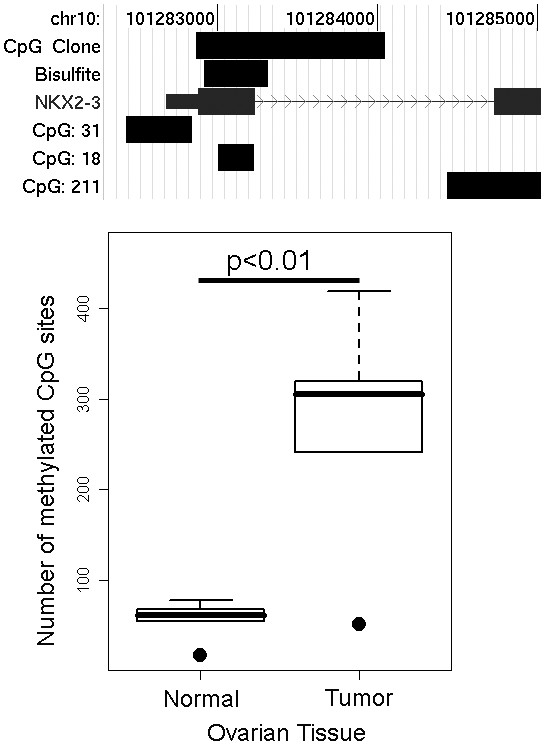
**Confirmation of increased methylation at a NKX2-3 gene CpG island in ovarian tumor by bisulfite sequencing. **a) Representation the 5' NKZ2-3 gene region showing the chromosomal location, associated CpG islands, region covered by the clone on the CGI array, region that was bisulfite sequenced, and the NKX2-3 gene itself. For the row labeled NKX2.3, the 5' untranslated region is indicated by the thin bar, the first and second exons are shown as thick bars, and the first intron is indicated by a thin line with overlaid arrow heads. b) Boxplot of the bisulfite sequencing results. The total number of methylated CpG sites in 47 clones from each of ten samples were averaged and plotted. The p-value for significance between the two means is shown (unpaired Welch's t-test).

The clones with significant differences in methylation between ovarian tissues included a gene of interest: NKX2-3. NKX2-3 is a member of the homeodomain-containing transcription factor family, and The NKX family members have been implicated in cell type-specific gene expression and regulation of cell differentiation. Our previous work identified another family of homeobox binding proteins, the HOXA cluster, as epigenetically silenced in breast cancer[25]. We therefore examined NKX2-3 further to verify our microarray results. To confirm the methylation changes found using the CGI microarrays, we measured methylation of the NKX2-3 CpG island that overlapped the clone on the CGI microarray by bisulfite sequencing. Figure 4a shows the position of the sequence queried on the CGI microarray relative to the region that was bisulfite sequenced, and the NKX2-3 gene sequence. Forty-seven bisulfite modified clones were sequenced from five stage III or IV tumors and five normal ovaries. The number of methylated CpG sites in each sample was counted and the result shown as a boxplot in Figure 4b. The difference in methylation detected between normal and tumor ovarian tissue by bisulfite sequencing was significant (p < 0.01, unpaired Welch's t-test).

### Re-expression of NKX2-3 by 5'-aza-deoxycytidine

The increased methylation of the NKX2.3 CpG island suggested epigenetic silencing of NKX2-3 expression that could be reversed by the demethlyating agent 5-aza-2'-deoxycytidine. To test this possibility, the ovarian cancer cell lines ES2 and OVCAR3 were treated with 5-aza-2'-deoxycytidine and re-expression of the NKX2-3 gene was measured by real time RT-PCR. As expected for an epigenetically silenced gene promoter, the 5-aza-2'-deoxycytidine treatment increased expression of NKX2-3 in a dose-dependent manner in both cell lines. NKX2-3 expression at 10 uM 5-aza-2'-deoxycytidine increased 7.2-fold in ES2 cells while the increase in OVCAR3 cells was 359-fold (Table 1).

**Table 1:** 5'-aza-deoxycytidine induced re-activation of NK2-3 gene expression in ovarian cancer cell lines

cell line	dose 5'-aza-dC (uM)	fold increase over untreated control cells
ES2	2	1.3
ES2	10	7.2
OVCAR3	2	55
OVCAR3	10	359

### Correlation of CpG methylation with gene expression

**Figure 5:**
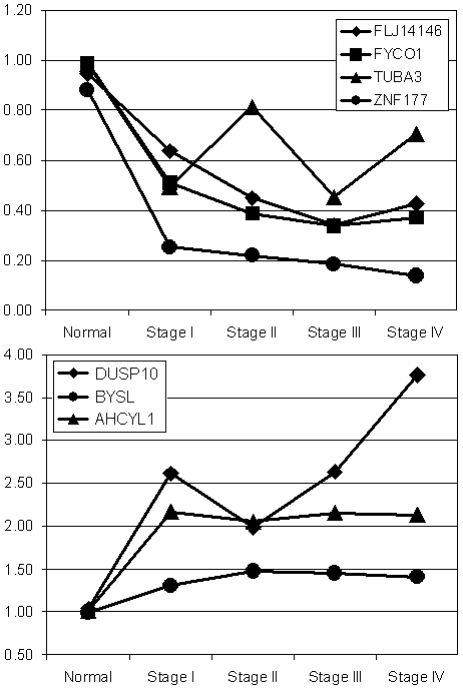
**Expression of genes with altered expression and CpG island methylation across ovarian cancer progression.** a) Expression profile of genes with increased CpG island methylation. b) Expression profile of genes with decreased CpG island methylation. Expression is graphed relative to the median of 4 normal samples and shown for all four stages of ovarian cancer.

Methylation of CpG islands associated with 5' gene promoters is associated with loss of expression, as was the case with NKX2-3 above. We therefore merged the CGI array data from stage III and IV ovarian tumors with gene expression data taken from a study by Hendrix et al. [26]. In their expression study, Hendrix et al. analyzed 99 individual ovarian tumors: 35 stage I, 11 stage II, 44 stage III, 9 stage IV, and 4 normal ovary samples on Affymetrix HG_U133A GeneChips (gene expression omnibus accession number: GSE6008). Of the 659 CpG-rich clones with DNA methylation changes in our CGI array data, 201 could be mapped to the U133A GeneChip using gene names: 126 were hyper-methylated with disease progression and 75 were hypo-methylated. Further selecting for the CpG islands most closely associated with 5' promoters (rank 1 or 2) resulted in 11 hypermethylated clones and 13 hypomethylated. Of the 11 clones with increased methylation of their CpG islands, four: FLJ14146, FYCO1, TUBA3, ZNF177 were expressed in normal ovary tissue and the expression of all four decreased with disease as would be predicted from the CpG methylation profile (Figure 5a). Of the 13 clones that lost methylation with disease progression, three were expressed in Stage III and IV tumors at higher levels than in normal ovary: AHCYL1, BYSL, and DUSP10 as suggested by their CpG methylation profiles (Figure 5b).

## Discussion

Genome-wide demethylation has long been thought to play a role in tumorigenesis[3]. Demethylation of the tumor cell genome has been observed in colon tumor cell lines and associated with chromosome instability in mouse stem cells[27,28]. The results reported here show both CpG methylation and demethylation occur with tumor progression in ovarian cancer. Demethylation occurred predominantly at repetitive elements (satellite and Alu repeats) of the genome. Demethylation of repetitive elements may contribute to genome instability and play a role in ovarian tumor progression[11]. The loss of methylation with disease progression showed a reversal between stages I and II, followed by a resumption of the trend in stage III in endometrial and serous, but not mucinous ovarian cancer. In addition to progressive loss of methylation at repetitive elements, our results show CpG methylation increased at hundreds of promoter CpG islands even as the genome was becoming demethylated on average.

The results suggest that the process of tumorigenesis differentiates between types of CpG-rich elements in order to simultaneously destabilize the genome while silencing tumor suppressor genes. In addition, the cumulative nature of the CpG methylation changes suggests that the stages of ovarian cancer are progressive with one arising from another. The exception to this observation was the histopathology-specific re-methylation of Alu and satellite CpG islands in stage II tumors relative to stage I tumors which reverted back to a de-methylated state in stage III tumors (Additional File 4). The observation raises the possibility that either stage II tumors are unique in their formation, or that there are two waves of demethylation, from benign to stage I, and then again in progression from stage II to stage III, during tumor progression.

To investigate the possible use of methylation patterns to identify ovarian cancer, we focused on comparing stage III serous papillary adenocarcinoma to normal and LMP tissue. Our results showed large numbers of CpG islands are hyper- and hypomethylated in stage III ovarian tumors relative to normal or LMP samples (Additional File 8). We were able to differentiate normal and LMP from stage III with a three-sequence classifier developed using the PAM algorithm with 87% sensitivity and 100% specificity on an independent test set of data (Figure 3). In addition, we used five other class prediction methods which had a high degree of agreement in both cross-validation of the training data set and prediction of the test data set (98–100% correct). The agreement between the various class prediction methods supports the likelihood that our methylation data could produce a classifier capable of differentiating between Normal and LMP samples and Stage III cancer. Furthermore, estimation of the cross-validated mis-classification error rate indicated that the classifier's performance was unlikely due to chance (p < 0.001).

The progressive nature of the methylation changes seen between stage III and normal or LMP samples suggests that the same changes, though smaller in magnitude, may be useful in detecting early (stage I) cancers using more sensitive methods for measuring altered CpG methylation. Early detection of ovarian cancer should improve patient treatment, and is the focus of current research using protein detection in blood samples[29]. Further development of the methylation profiles identified here may provide an additional variable that could complement protein expression to further improve detection of early cancer. The demonstration that mutations in tumor DNA are detectable in the blood supports the possibility of detecting methylation events in the blood of women with ovarian cancer in order to achieve an earlier diagnosis[30].

To validate the CGI microarray results, we confirmed hypermethylation at a NKX2-3 CpG island in ovarian tumor samples relative to normal tissue using bisulfite sequencing. In addition, NKX2-3, was reactivated in two ovarian tumor cell lines following treatment with the demethlyating agent 5'-aza-2'deoxycytidine. Although we did not measure the methylation status of the NKX2-3 CpG island in the cell lines, re-expression of the gene following treatment with 5'-aza-2'deoxycytidine and confirmation of methylation in patient samples suggests that NKX2-3 is epigenetically silenced during ovarian tumorigenesis. As a further confirmation of the CpG island microarrays, we identified seven clones whose methylation profile predicted their loss or gain in expression with disease progression by analyzing our methylation data in conjunction with Wu et al.'s expression data. These results demonstrate that CpG island microarrays can be used to identify novel targets of epigenetic control in tumor samples.

## Conclusion

We found patterns of DNA methylation that distinguish tumor samples from benign tissue, and used this data to discover genes affected by epigenetic regulation. We showed that the changes in methylation are cumulative with increasing stage, thus the methylation changes may provide a marker for early detection of disease. Our results extend previous studies that suggested simultaneous hypo- and hyper-methylation occurred with tumor progression by measuring both in the same tumor cells.

## Abbreviations

LMP: low malignant potential; FDR: False Discovery Rate; ANOVA: analysis of variance; PAM: prediction analysis of microarrays; SVM: support vector machines.

## Competing interests

There are no financial competing interests to disclose. The authors state that no financial support has been provided by any organization that stands to gain or lose financially from publication of the work presented here. The authors do not hold stock in any organization that may benefit from this publication, nor are any patents relating to this work held or applied for.

## Authors' contributions

GSW: experiment conception and design, data analysis, manuscript preparation; BWF: experiment conception and design, data analysis; NH: data generation; KDG: experiment design and critical discussion, samples; FED: experiment conception and design, data analysis; SR: critical discussion, experiment conception and design, data analysis, samples.

## Pre-publication history

The pre-publication history for this paper can be accessed here:



## Supplementary Material

Additional file 1 Three M versus A plots (intensity ratios *M *= (*R*/*G*) versus average intensities *A *= (R*G)/2 for three representative hybridizations. The sample ID is shown in the upper right of each graph. Click here for file

Additional file 2 **Table listing all samples with their stage, histopathology, and grade.** Click here for file

Additional file 3 **The 659 CpG-rich clones significant (p < 0.01, 1.5-fold change) methylation differences between any stage.** All data normalized to the median of the 10 normal ovarian tissue samples. Average methylation for each stage is shown. Clones associated with a gene (rank 1–4) have the gene's accession number listed. Click here for file

Additional file 4 **The cumulative loss of DNA methylation with ovarian cancer progression reverts temporarily in endometroid and serous papillary adenocarcinomas, but not mucinous adenocarcinomas.** The 659 CpG-rich clones with significant (p < 0.01, 1.5-fold change) methylation differences between any two stages are graphed by histopathology and tumor stage. A reversion to a more normal methylation state can be seen for the sequences with overall loss of methylation in the progression from stage I to stage II in the endometroid and serous papillary adenocarcinomas. Each CpG-rich clone is represented by one line. Lines are colored by their average methylation in Stage IV papillary serous adenocarcinoma relative to the median of the ten normal samples; blue indicates loss of methylation, and red indicates gain of methylation. Click here for file

Additional file 5 **Cumulative loss and gain of DNA methylation in the progression from low to high grade ovarian cancer.** The 659 CpG-rich clones with significant (p < 0.01, 1.5-fold change) changes in methylation between any two stages of cancer are graphed by grade. Each CpG-rich clone is represented by one line. Lines are colored by their average methylation in grade 3 tumors relative to the median of the ten normal samples; blue indicates loss of methylation, and red indicates gain of methylation. The average methylation of the normal and low malignant potential samples is shown combined as "benign". Click here for file

Additional file 6 **Data for all CpG-rich clones on the CGI microarray with replicates spots averaged. All data normalized to the median of the 10 normal ovarian tissue samples.** Average methylation for each stage is shown. Clones associated with a gene (rank 1–4) have the gene's accession number listed. Click here for file

Additional file 7 **The results of the BRB ArrayTools analysis are shown, including cross-validation results, and results on the test for each of the five class prediction methods, as well as the list of the 911 sequences that compose the classifier.** An additional sheet shows the methylation data for the 911 sequences across all disease stages with all data normalized to the median of the ten normal ovarian tissue samples. Average methylation for each stage is shown. Clones associated with a gene (rank 1–4) have the gene's accession number listed. Click here for file

Additional file 8 **The 373 CpG-rich clones used by the class prediction methods with 1.5-fold changes in DNA methylation between normal and LMP, and stage III samples graphed by tumor stage.** Each CpG-rich clone is represented by one line. Lines are colored by their average methylation in Stage IV relative to the median of the ten normal samples; blue indicates loss of methylation, and red indicates gain of methylation. Click here for file
