# Supplementary figures and images for: DNA methylation changes in ovarian cancer are cumulative with disease progression and identify tumor stage
Source: BMC Med Genomics. 2008 Sep 30;1:47. doi: 10.1186/1755-8794-1-47 (PMC2566571; doi:10.1186/1755-8794-1-47)

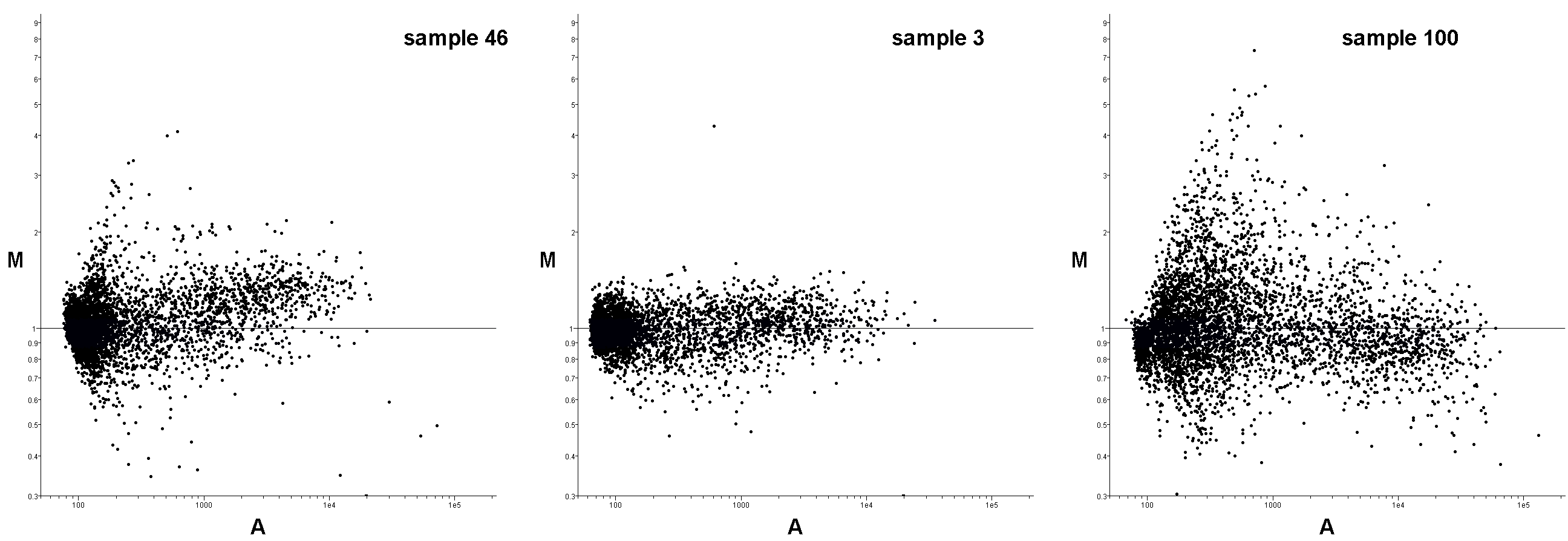

Supplement: Additional file 1 — Three M versus A plots (intensity ratios M = (R/G) versus average intensities A = (R*G)/2 for three representative hybridizations. The sample ID is shown in the upper right of each graph. [file 1755-8794-1-47-S1.tiff]

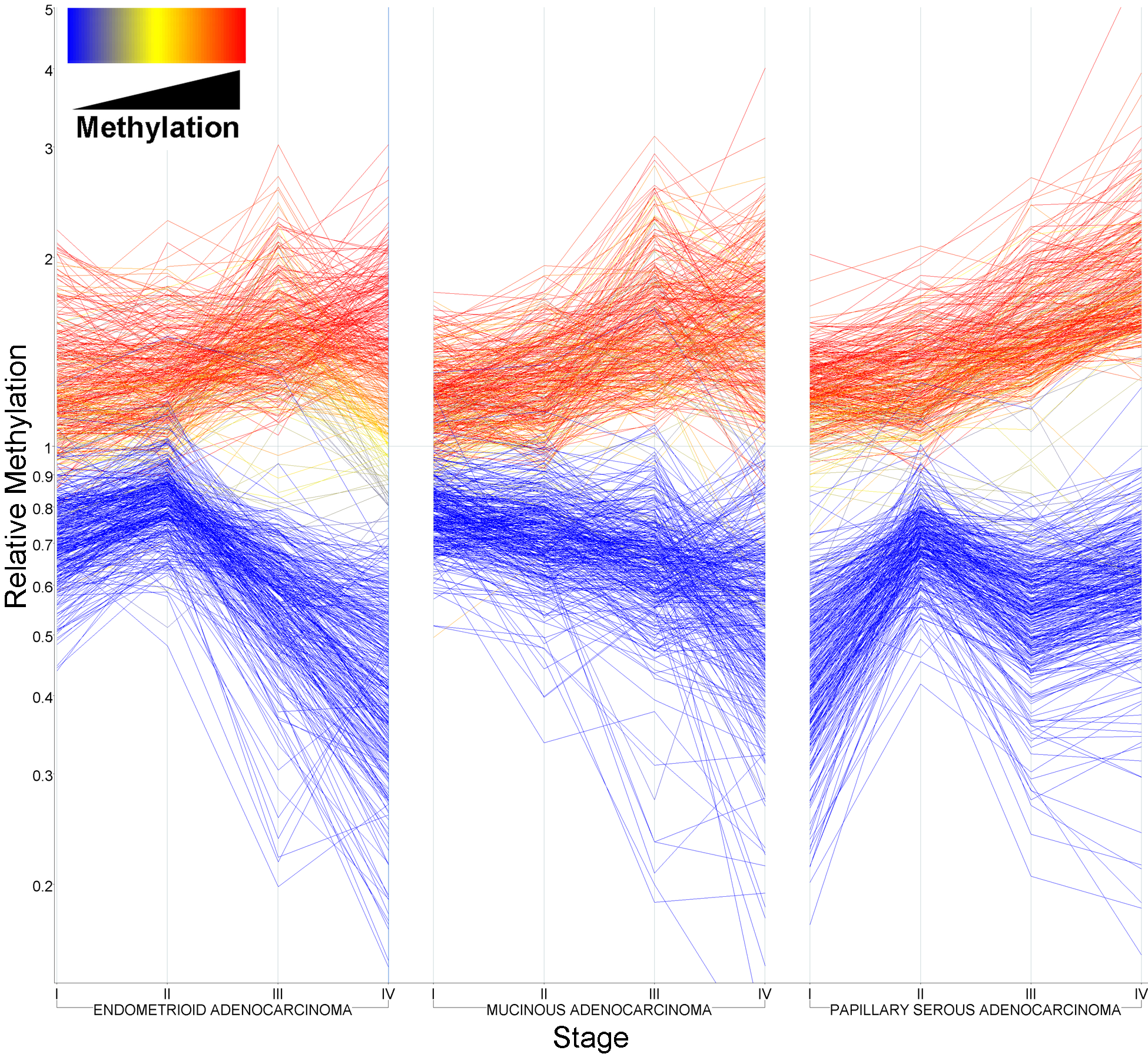

Supplement: Additional file 4 — The cumulative loss of DNA methylation with ovarian cancer progression reverts temporarily in endometroid and serous papillary adenocarcinomas, but not mucinous adenocarcinomas. The 659 CpG-rich clones with significant (p < 0.01, 1.5-fold change) methylation differences between any two stages are graphed by histopathology and tumor stage. A reversion to a more normal methylation state can be seen for the sequences with overall loss of methylation in the progression from stage I to stage II in the endometroid and serous papillary adenocarcinomas. Each CpG-rich clone is represented by one line. Lines are colored by their average methylation in Stage IV papillary serous adenocarcinoma relative to the median of the ten normal samples; blue indicates loss of methylation, and red indicates gain of methylation. [file 1755-8794-1-47-S4.tiff]

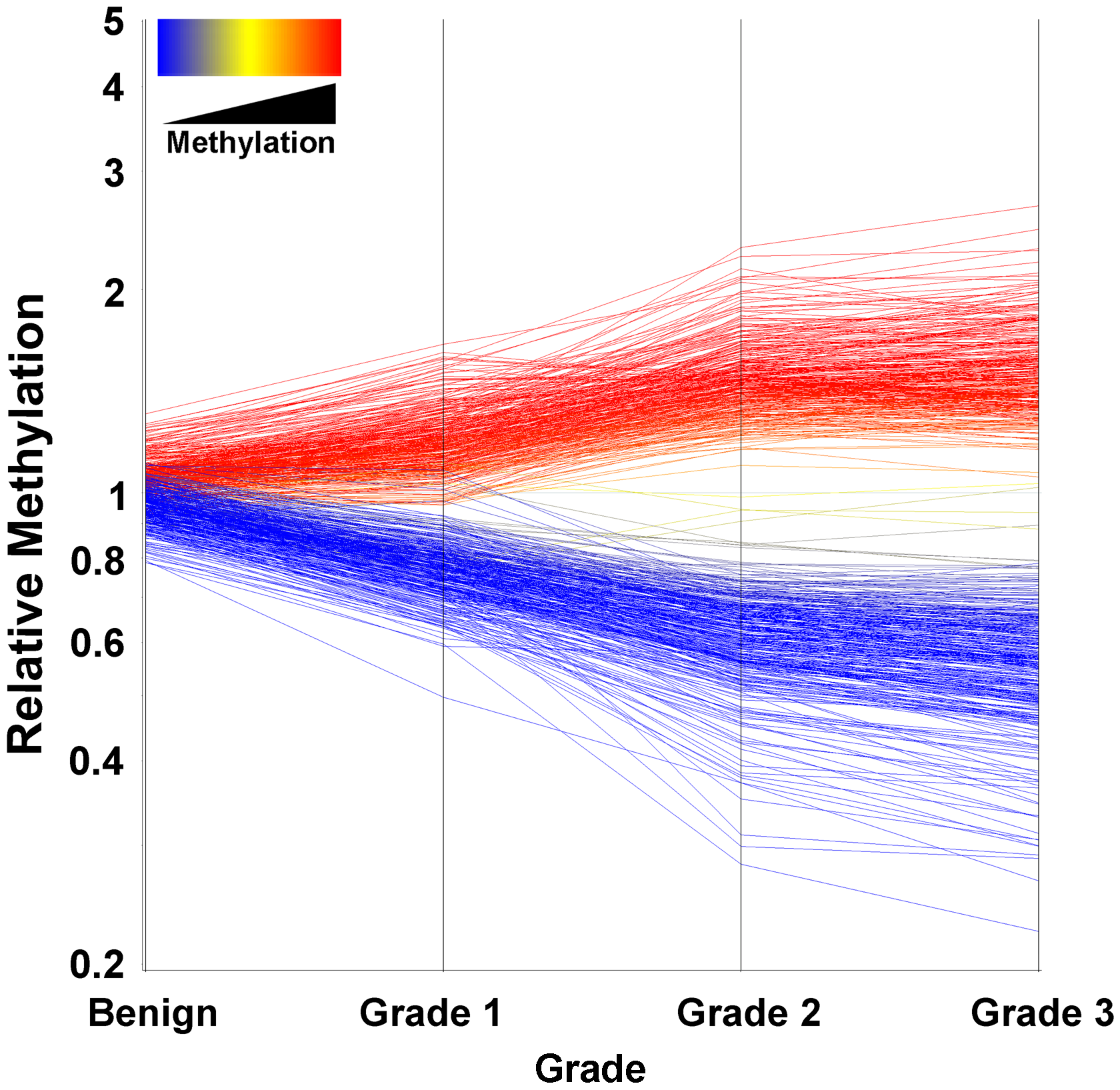

Supplement: Additional file 5 — Cumulative loss and gain of DNA methylation in the progression from low to high grade ovarian cancer. The 659 CpG-rich clones with significant (p < 0.01, 1.5-fold change) changes in methylation between any two stages of cancer are graphed by grade. Each CpG-rich clone is represented by one line. Lines are colored by their average methylation in grade 3 tumors relative to the median of the ten normal samples; blue indicates loss of methylation, and red indicates gain of methylation. The average methylation of the normal and low malignant potential samples is shown combined as "benign". [file 1755-8794-1-47-S5.tiff]

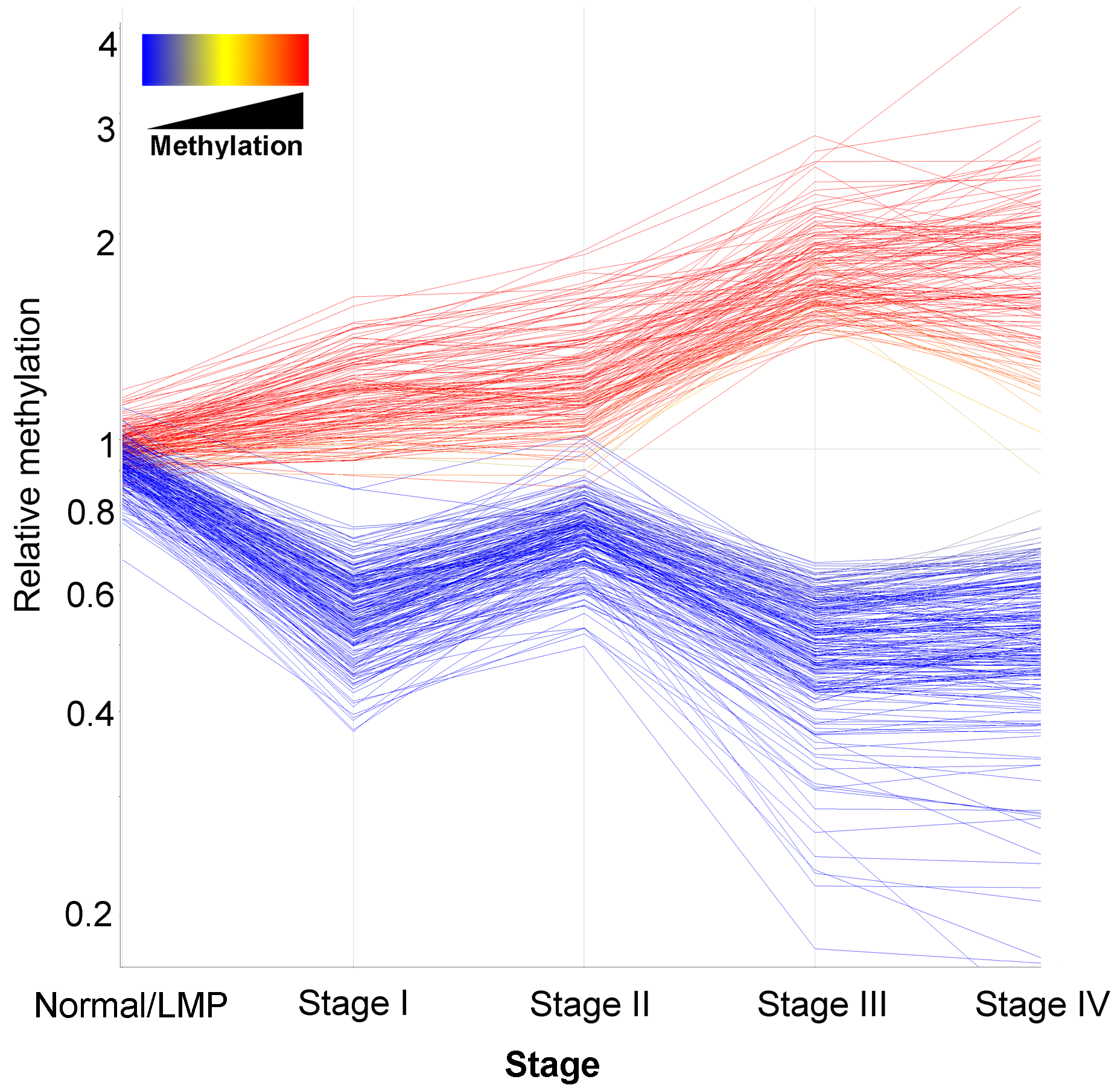

Supplement: Additional file 8 — The 373 CpG-rich clones used by the class prediction methods with 1.5-fold changes in DNA methylation between normal and LMP, and stage III samples graphed by tumor stage. Each CpG-rich clone is represented by one line. Lines are colored by their average methylation in Stage IV relative to the median of the ten normal samples; blue indicates loss of methylation, and red indicates gain of methylation. [file 1755-8794-1-47-S8.tiff]
